# Effects of creatine loading on electromyographic fatigue threshold during cycle ergometry in college-aged women

**DOI:** 10.1186/1550-2783-4-20

**Published:** 2007-11-26

**Authors:** Abbie E Smith, Ashley A Walter, Trent J Herda, Eric D Ryan, Jordan R Moon, Joel T Cramer, Jeffrey R Stout

**Affiliations:** 1 Metabolic and Body Composition Laboratory, Department of Health and Exercise Science, University of Oklahoma, Norman, OK 73019, USA; 2 Biophysics Laboratory, Department of Health and Exercise Science, University of Oklahoma, Norman, OK 73019, USA

## Abstract

The purpose of this study was to examine the effects of 5 days of Creatine (Cr) loading on the electromyographic fatigue threshold (EMG_FT_) in college-aged women. Fifteen healthy college-aged women (mean ± SD = 22.3 ± 1.7 yrs) volunteered to participate in this double-blind, placebo-controlled study and were randomly placed into either placebo (PL – 10 g of flavored dextrose powder; n = 8) or creatine (Cr – 5 g di-creatine citrate plus 10 g of flavored dextrose powder; n = 7; *Creatine Edge*, FSI Nutrition) loading groups. Each group ingested one packet 4 times per day (total of 20 g/day) for 5 days. Prior to and following supplementation, each subject performed a discontinuous incremental cycle ergometer test to determine their EMG_FT _value, using bipolar surface electrodes placed on the longitudinal axis of the right vastus lateralis. Subjects completed a total of four, 60 second work bouts (ranging from 100–350 W). The EMG amplitude was averaged over 10 second intervals and plotted over the 60 second work bout. The resulting slopes from each successive work bouts were used to calculate EMG_FT_. A two-way ANOVA (group [Cr vs. PL] × time [pre vs. post]) resulted in a significant (*p = 0.031*) interaction. Furthermore, a dependent samples t-test showed a 14.5% ± 3.5% increase in EMG_FT _from pre- to post-supplementation with Cr (*p = 0.009*), but no change for the PL treatment *(-*2.2 ± 5.8%; *p = 0.732*). In addition, a significant increase (1.0 ± 0.34 kg; *p = 0.049*) in weight (kg) was observed in the Cr group but no change for PL (-0.2 kg ± 0.2 kg). These findings suggest that 5 days of Cr loading in women may be an effective strategy for delaying the onset of neuromuscular fatigue during cycle ergometry.

## Background

In a series of electromyographic (EMG) fatigue studies, Moritani et al. [1,2] demonstrated an increase in EMG activity during incremental cycling exercise. It has been suggested that the rise in electrical activity is a result of progressive recruitment of additional motor units (MU) and/or an increase in the firing frequency of MUs that have already been recruited [3]. An exercise-induced decrease in intramuscular pH, due to increases in hydrogen ions [H^+^], may interfere with the excitation-contraction coupling process of skeletal muscle, which, in turn, may lead a to decrease in power output and fatigue [4]. Thus, if power output is to be maintained, either additional MUs must be recruited or the firing rates of the already active MUs must increase. In either case, a rise in EMG amplitude with power output takes place due to increases in muscle activation.

The physiological mechanism responsible for the increase in EMG amplitude over time during a fatiguing task is unknown. Three potential mechanisms, however, include the accumulation of metabolic by-products (lactate, hydrogen ions (H^+^), inorganic phosphate (Pi), and ammonia), the depletion of stored energy substrates (ATP, phosphocreatine (PCr), and glycogen) and/or impaired muscle cation (potassium (K^+^), sodium (Na^+^) and calcium (Ca^2+^) regulation [5-7]. Several investigations have used surface electromyography to characterize the fatigue-induced increase in EMG amplitude, as well as to identify the power output associated with the onset of neuromuscular fatigue during cycle ergometry [2,8-11]. Furthermore, surface EMG has been shown to be an acceptable method for non-invasive assessment of muscle fatigue of active muscles [12,13].

Matsumoto et al. [9] and Moritani et al. [2] have proposed an incremental cycle ergometer test utilizing fatigue curves to identify the maximal power output at which an individual can maintain without evidence of fatigue, called the electromyographic fatigue threshold (EMG_FT_). The EMG_FT _test is an adaptation to deVries original monopoloar physical working capacity at the fatigue threshold (PWC_FT_) test [14], using a bipolar supramaximal protocol, which involves determining the rate of rise in electrical activity from the vastus lateralis during four, 60 second work bouts on a cycle ergometer, with varying power outputs. The four power outputs are then plotted as a function of four EMG slope coefficients, with the y-intercept defined as the electromyographic fatigue threshold (EMG_FT_).

Matsumoto et al[9] described the EMG_FT _as the highest intensity sustainable on a cycle ergometer without signs of neuromuscular fatigue. In addition, Moritani et al. [2] suggested a strong physiological link between myoelectrical changes at fatigue and anaerobic threshold. Furthermore, the EMG_FT _method has been reported as a valid and reliable technique for examining the transition from aerobic to anaerobic metabolism during exercise [8,12,13].

Harris et al. [15] were the first to demonstrate a 20% increase in muscle creatine (Cr) after ingestion of 20 g/day of Cr (four doses of 5 g) (Cr) for 5 days, with a 30% increase as phosphocreatine (PCr). The effect Cr supplementation on anaerobic activities, utilizing anaerobic energy reserves (adenosine triphosphate (ATP) PCr) has received a large amount of attention. It has been suggested that skeletal muscle PCr may serve as a temporal energy buffer as well as a modulator of glycolysis, and that, if increased via Cr supplementation, may influence neuromuscular fatigue [11,16]. Although Cr has been reported to be most effective in anaerobic activities, some evidence suggests that Cr may serve as an effective temporal energy buffer [17,18] and may enhance aerobic activities[11,19,20].

Although women have been underrepresented in the Cr literature, recent studies suggest that there are no gender differences in the amount of increase of total creatine (TCr) following loading [15,21] nor are there differences in high intensity exercise performance [22]. In addition, Stout et al. [11] demonstrated improvements in neuromuscular fatigue after 5 days of Cr loading (4 × 5 g·d^-1^) in trained female athletes, with a significant (p < 0.05) increase in PWC_FT_. To date, however, no studies have examined the effects of Cr loading on EMG_FT _in women. Therefore, the purpose of this study was to examine the effects of 5 days of Cr loading on EMG_FT _in college-aged women.

## Methods

### Participants

Fifteen healthy, recreationally trained (1–5 hours/week) college-aged women volunteered to participate in this investigation (Table 1). All procedures were approved by the University of Oklahoma Institutional Review Board for Human subjects and written informed consent was obtained from each participant prior to any testing.

**Table 1:** Age, height, and body weight of the participants at baseline.

	Age (years)	Height (cm)	Weight (kg)
Creatine (n = 7)	22.4 ± 0.5	168.8 ± 1.3	66.0 ± 3.6
Placebo (n = 8)	22.3 ± 0.7	170.3 ± 2.9	64.7 ± 2.9

Values expressed as mean ± SE

### Supplementation

This was a randomized, double-blind, placebo-controlled design. Participants were randomly assigned to either the placebo (PL – 10 g of flavored dextrose powder per packet; n = 9) or creatine (Cr – 5 g di-creatine citrate plus 10 g of flavored dextrose powder per packet; n = 7) (Creatine Edge, FSI Nutrition Inc. Omaha, NE) groups. Both treatments were effervescent powders, pre-packaged to be identical in taste and appearance, and were dissolved in 8–12 oz of water. Each group ingested one packet 4 times per day at regular intervals (every 3–4 hours) for 5 days (total of 20 g/day). Throughout the duration of the study, participants were asked to refrain from physical activity and caffeine consumption, for at least 24 hours prior to testing.

### Electromyography

Pre-gelled bipolar (2.54 cm center-to-center) surface electrodes (Ag-Ag Cl, Quinton Quick Prep, Quinton Instruments Co., Bothell, WA) were placed on the right thigh over the lateral portion of the vastus lateralis muscle, midway between the greater trochanter and the lateral condyle of the femur [1,2,9-11,23]. A single reference electrode was placed over the spinous process of the 7^th ^cervical vertibrae. Interelectode impedance was kept below 2,000 Ω by careful abrasion of the skin. The raw EMG signals were pre-amplified (gain × 1,000) using a differential amplifier (EMG 100C, Biopac Systems, Inc., Santa Barbara, CA), sampled at 1,000 Hz and bandpass filtered from 10–500 Hz (zero-lag 8^th ^order Butterworth filter). All EMG amplitude values were stored on a personal computer (Dell Inspiron 8200, Dell, Inc., Round Rock, TX) and analyzed off-line using custom written software (LabVIEW v 7.1, National Instruments, Austin, TX).

### Determination of EMG_FT_

**Figure 1:**
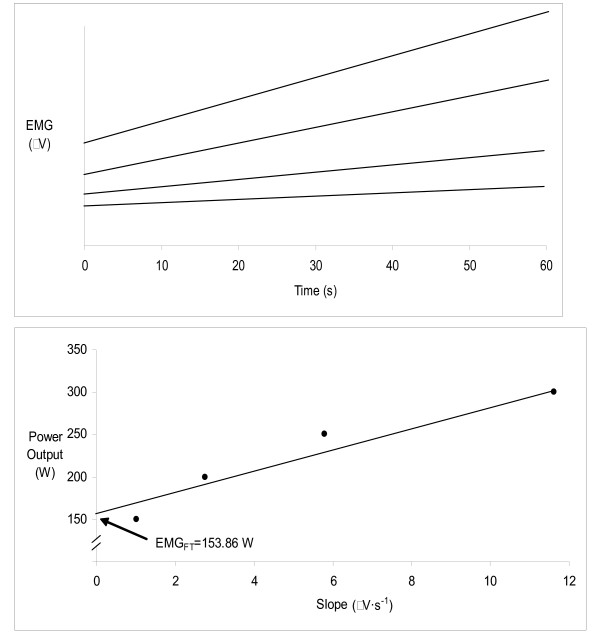
a. The relationship between EMG amplitude and time for the four power outputs used in the EMG_FT _test for subject 5C. The greatest slope was a result from the highest power output. b. The relationship for the power outputs versus slope coefficients with the y-intercept defined as the EMG_FT _for subject 5C.

The EMG_FT _was determined using the EMG amplitude values from the vastus lateralis muscle while cycling on an electronically braked cycle ergometer (Quinton Corival 400), with the seat height adjusted at near full extension of the legs while pedaling. After a 5 minute warm-up (50 W), the participants performed four, 60 second incrementally ascending workloads (100 W–350 W) at a set cadence of 70 rpm[8,9,23]. The initial workload was determined by fitness level [9,23]. Adequate rest was given between bouts to allow for participants' heart rate to drop within 10 beats of their resting heart rate (averaging 10–15 minutes). The rate of rise in EMG amplitude values (EMG slope) from four fatiguing power outputs were plotted over 60 seconds (Figure 1a). The EMG slope values for each of the four power outputs were then plotted (Figure 1b) to determine EMG_FT_. The line of best fit was extrapolated to the y-axis and the power output at which it intersected was defined as the EMG_FT_. The participants completed the EMG_FT _protocol three times; familiarization (day 1), baseline (day 3) and post supplementation (day 9), respectively.

Test-retest reliability for the EMG_FT _protocol was determined at the University of Oklahoma by using 10 healthy male and female participants measured 7–8 days apart. The intraclass correlation coefficient (ICC) was 0.935 (SEM 5.03 W), which was higher than previously reported for the vastus lateralis (ICC = 0.65) by Pavlet et al. (1993). In addition, there was no significant difference (p > 0.05) between the mean EMG_FT _values from pre- (172.17 ± 14.12 W) to post- (165.95 ± 13.41 W).

### Data Analysis

A two-way ANOVA (group [Cr vs. PL] × time [pre vs. post]) was used to identify any group by time interaction. When appropriate, follow up dependent t-test analyses were run on pre to post-EMG_FT _and body weight (BW) values. Furthermore, an independent samples t-test was used to recognize statistical significance among pre-supplementation variables (EMG_FT _and BW). All data are reported as mean ± S.E.

## Results

**Figure 2:**
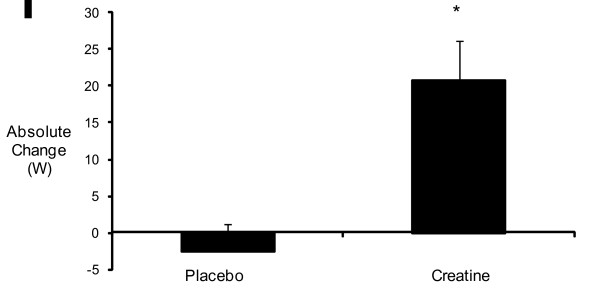
The effect of creatine supplementation on Electromyographic Fatigue Threshold (EMG_FT_; W) from pre- to post-treatment. Values are presented as mean ± SD. *The post-supplemented mean EMG_FT _is significantly (p < 0.01) higher than the pre-supplemented mean EMG_FT_.

Table 2 shows the mean and standard error values for EMG_FT _and BW. A two-way ANOVA resulted in a significant interaction (*p = 0.024*) for group and time. There was a significant (*p = 0.031*) difference in absolute change from pre- to post-treatment in EMG_FT _between the Cr and the PL treatment groups (Fig 2). In addition, dependent-samples t-test indicated a 14.5% ± 3.5% increase in EMG_FT _from pre- to post-supplementation for the Cr treatment (*p = 0.009*), but no significant change for the PL treatment (*-*2.2 ± 5.8%; *p = 0.732*) (Table 2). Cr supplementation resulted in a significant 1.0 kg ± 0.34 kg increase (*p = 0.049*) in BW (Fig 2b) from pre- to post-supplementation, but no change in the PL treatment group (-0.2 kg ± 0.2 kg).

**Table 2:** Mean and standard error (SE) values for electromyographic fatigue threshold and body weight for the pre- and post-supplementation trials with either the placebo or creatine.

	Electromographic Fatigue Threshold (W)		Body Weight (kg)	
	
	Placebo	Creatine	Placebo	Creatine
Pre-supplementation	152.3 ± 11.6	121.9 ± 10.9	64.7 ± 2.9	66.0 ± 3.6
Post-supplementation	149.5 ± 9.3	142.6 ± 10.5*	64.9 ± 2.9	66.8 ± 3.7*

*p < 0.05 for pre- to post-difference

Additionally, independent samples t-tests demonstrated no significant differences between either pre-supplementation variables, p > 0.05 for EMGFT and BW respectively.

## Discussion

Several studies have reported 4 to7 days of Cr loading is effective for enhancing anaerobic performance during repeated sprint cycling [24-27], resistance training [28,29], and anaerobic treadmill running [30]. In addition, a few studies have demonstrated Cr loading to be an effective ergogenic aid for supramaximal workloads (>120% VO_2 max_) on a cycle ergometer [27,31-33]. The increase in exercise performance is believed to be attributed to an increase in intramuscular stores of total PCr [15,34]. A few investigators [11,16,33] have suggested that increasing skeletal muscle PCr from Cr loading may improve supramaximal cycle ergometry performance by decreasing the reliance on anaerobic glycolysis for energy. In theory, decreasing the contribution of anaerobic glycolysis to meet energy demands during supramaximal workloads may reduce intramuscular lactate and H^+ ^accumulation and, in turn, delay the onset of fatigue. In addition, Cr loading may also delay fatigue by increasing the rate of PCr resynthesis between intense exercise bouts, enhance buffering capacity, and provide greater shuttling of mitochondrial ATP into the cytoplasm [15,18,35].

McClaren et al. [5] suggested that a decrease in muscle pH, as a result of the accumulation of intramuscular H^+ ^or an increase in intracellular and extracellular ammonia, may be responsible for fatigue-induced increases in motor unit recruitment and the corresponding increase in EMG amplitude. In agreement, Taylor et al. [36] also found that, for incremental cycle ergometry, the accumulation of plasma lactate and ammonia was associated with an increase in EMG amplitude. Therefore, there is evidence to suggest that a significant reliance on anaerobic glycolysis leads to an increase in EMG amplitude from the working muscles, as a result of changes in muscle and blood lactate levels and the corresponding decrease in skeletal muscle pH [2,36].

Moritani et al. [2] speculate that the EMG_FT _may be closely associated with a steady state of lactate metabolism and H+ accumulation in the active muscle. The level of exercise just below the point at which the bicarbonate buffering system can no longer maintain a homeostatic environment, due to an increase in H^+^, has been described as anaerobic threshold (AT) [37]. In support, deVries [14] and Matsumoto et al. [9] demonstrated a close relationship between AT and the EMG_FT _test. Furthermore, Matsumoto et al. [9] reported a significant relationship (r = 0.823; p < 0.01) between AT and EMG_FT _with a similar subject sample of college-age women. In addition, Wasserman et al. [38] and Sirotic et al. [39] have demonstrated a strong relationship between AT and the ability of the cardiovascular system to supply oxygen during exercise, coupled with an improvement in performance. The results of our study indicated a 14.5% increase in EMG_FT _after 5 days of Cr loading, which are very similar to Nelson et al. [20] who reported a 15% increase in AT in men and women from 7 days of Cr loading using a graded exercise test (GXT) on a cycle ergometer. In addition, Stout et al. [11] using a similar cohort of college-age women, reported a 13% increase in neuromuscular fatigue threshold during a GXT. While our results were similar to Nelson et al. [20] and Stout et al. [11] at increasing estimated fatigue thresholds, the present study utilized a supramaximal discontinuous protocol, compared to a submaximal incremental test, to estimate anaerobic and neuromuscular fatigue thresholds. To our knowledge this is the first study to report the effect of Cr loading on the supramaximal EMG_FT _assessment, in women.

The EMG_FT _theoretically represents the highest power output that can be sustained, during cycle ergometry, for an extended period of time without signs of neuromuscular fatigue [9]. The primary findings of this study suggest that Cr loading may significantly increase the EMG_FT _in women, and thereby increase the power output that can be maintained on a cycle ergometer for an extended period of time without exhaustion. Additionally, in contrast to previous studies using women [34,40,41], the results of the present study for BW (Table 1) showed a significant increase from pre- to post-Cr loading. Although there was a significant increase in BW, the change (<0.8 kg) may have represented day-to-day fluctuations.

In conclusion, Cr loading in college-age women resulted in a significant increase in EMG_FT _(14.5%) when compared to the placebo group (-2.5%). The increase in EMG_FT _may have been due to the increased intramuscular PCr levels, attenuating the contribution of anaerobic glycolysis to ATP resynthesis [11,16,33,42,43]. Future studies that directly measure changes in skeletal muscle PCr and AT measures (EMG_FT_, lactate, ventilatory and ammonia thresholds) may be warranted to confirm these results.

## Competing interests

The author(s) declare that they have no competing interests.

## Authors' contributions

All authors contributed equally to this work. All authors have read and approved the final manuscript.

## References

[B1] Moritani T, Muro M, Nagata A (1986). Intramuscular and surface electromyogram changes during muscle fatigue. J Appl Physiol.

[B2] Moritani T, Takaishi T, Matsumoto T (1993). Determination of maximal power output at neuromuscular fatigue threshold. J Appl Physiol.

[B3] Kamen G, Caldwell GE (1996). Physiology and interpretation of the electromyogram. J Clin Neurophysiol.

[B4] Fitts RH, Holloszy JO (1976). Lactate and contractile force in frog muscle during development of fatigue and recovery. The American journal of physiology.

[B5] McClaren DP, Gibson H, Parry-Billings M, Edwards RHT (1989). A review of metabolic and physiological factors in fatigue. Exerc Sport Sci Rev.

[B6] Green HJ (1998). Cation pumps in skeletal muscle: potential role in muscle fatigue. Acta physiologica Scandinavica.

[B7] McKenna MJ (1992). The roles of ionic processes in muscular fatigue during intense exercise. Sports medicine (Auckland, NZ.

[B8] Maestu J, Cicchella A, Purge P, Ruosi S, Jurimae J, Jurimae T (2006). Electromyographic and neuromuscular fatigue thresholds as concepts of fatigue. Journal of strength and conditioning research / National Strength & Conditioning Association.

[B9] Matsumoto T, Ito K, Moritani T (1991). The relationship between anaerobic threshold and electromyographic fatigue threshold in college women. European journal of applied physiology and occupational physiology.

[B10] Pavlat DJ, Housh TJ, Johnson GO, Schmidt RJ, Eckerson JM (1993). An examination of the electromyographic fatigue threshold test. European journal of applied physiology and occupational physiology.

[B11] Stout J, Eckerson J, Ebersole K, Moore G, Perry S, Housh T, Bull A, Cramer J, Batheja A (2000). Effect of creatine loading on neuromuscular fatigue threshold. J Appl Physiol.

[B12] Hug F, Laplaud D, Savin B, Grelot L (2003). Occurrence of electromyographic and ventilatory thresholds in professional road cyclists. European journal of applied physiology.

[B13] Lucia A, Sanchez O, Carvajal A, Chicharro JL (1999). Analysis of the aerobic-anaerobic transition in elite cyclists during incremental exercise with the use of electromyography. British journal of sports medicine.

[B14] deVries HA, Moritani T, Nagata A, Magnussen K (1982). The relation between critical power and neuromuscular fatigue as estimated from electromyographic data. Ergonomics.

[B15] Harris RC, Soderlund K, Hultman E (1992). Elevation of creatine in resting and exercised muscle of normal subjects by creatine supplementation. Clin Sci (Lond).

[B16] Volek JS, Kraemer WJ (1996). Creatine Supplementation: its effect on human muscular performance and body composition. J Strength Cond Res.

[B17] Bogdanis GC, Nevill ME, Boobis LH, Lakomy HK (1996). Contribution of phosphocreatine and aerobic metabolism to energy supply during repeated sprint exercise. J Appl Physiol.

[B18] Greenhaff PL, Bodin K, Soderlund K, Hultman E (1994). Effect of oral creatine supplementation on skeletal muscle phosphocreatine resynthesis. The American journal of physiology.

[B19] Aoki MS, Gomes RV, Raso V (2004). Creatine supplementation attenuates the adverse effect of endurance exercise on subsequent resistance exercise performance. Med Sci Sports Exerc.

[B20] Nelson AG, Day R, Glickman-Weiss EL, Hegsted M, Kokkonen J, Sampson B (2000). Creatine supplementation alters the response to a graded cycle ergometer test. European journal of applied physiology.

[B21] McKenna MJ, Morton J, Selig SE, Snow RJ (1999). Creatine supplementation increases muscle total creatine but not maximal intermittent exercise performance. J Appl Physiol.

[B22] Stout JR, Eckerson JM, Housh TJ, Ebersole KT (1999). The effects of creatine supplementation on anaerobic working capacity.. J Strength Cond Res.

[B23] Housh TJ, deVries HA, Johnson GO, Housh DJ, Evans SA, Stout JR, Evetovich TK, Bradway RM (1995). Electromyographic fatigue thresholds of the superficial muscles of the quadriceps femoris. European journal of applied physiology and occupational physiology.

[B24] Cooke WH, Grandjean PW, Barnes WS (1995). Effect of oral creatine supplementation on power output and fatigue during bicycle ergometry. J Appl Physiol.

[B25] Jacobs I, Bleue S, Goodman J (1997). Creatine ingestion increases anaerobic capacity and maximum accumulated oxygen deficit. Canadian journal of applied physiology = Revue canadienne de physiologie appliquee.

[B26] Birch R, Noble D, Greenhaff PL (1994). The influence of dietary creatine supplementation on performance during repeated bouts of maximal isokinetic cycling in man. European journal of applied physiology and occupational physiology.

[B27] Tarnopolsky MA, MacLennan DP (2000). Creatine monohydrate supplementation enhances high-intensity exercise performance in males and females. International journal of sport nutrition and exercise metabolism.

[B28] Vandenberghe K, Goris M, Van Hecke P, Van Leemputte M, Vangerven L, Hespel P (1997). Long-term creatine intake is beneficial to muscle performance during resistance training. J Appl Physiol.

[B29] Volek JS, Kraemer WJ, Bush JA, Boetes M, Incledon T, Clark KL, Lynch JM (1997). Creatine supplementation enhances muscular performance during high-intensity resistance exercise. Journal of the American Dietetic Association.

[B30] Earnest CP, Almada AL, Mitchell TL (1997). Effects of creatine monohydrate ingestion on intermediate duration anaerobic treadmill running to exhaustion. J Strength Cond Res.

[B31] Balsom PD, Ekblom B, Soderlund K, Sjodin B, Hultman E (1993). Creatine supplementation and dynamic high-intensity exercise. Scand J Med Sci Sports.

[B32] Casey A, Constantin-Teodosiu D, Howell S, Hultman E, Greenhaff PL (1996). Creatine ingestion favorably affects performance and muscle metabolism during maximal exercise in humans. The American journal of physiology.

[B33] Prevost MC, Nelson AG, Morris GS (1997). Creatine supplementation enhances intermittent work performance. Research quarterly for exercise and sport.

[B34] Eckerson JM, Stout JS, Moore GA, Stone NJ, Nishimura K, Tamura K (2004). Effect of two and five days of creatine loading in anerobic working capacity in women. J Strength Cond Res.

[B35] Hultman E, Soderlund K, Timmons JA, Cederblad G, Greenhaff PL (1996). Muscle creatine loading in men. J Appl Physiol.

[B36] Taylor AD, Bronks R, Bryant AL (1997). The relationship between electromyography and work intensity revisited: a brief review with references to lacticacidosis and hyperammonia. Electromyography and clinical neurophysiology.

[B37] Wasserman K, Whipp BJ, Koyl SN, Beaver WL (1973). Anaerobic threshold and respiratory gas exchange during exercise. J Appl Physiol.

[B38] Wasserman K, Koike A (1992). Is the anaerobic threshold truly anaerobic?. Chest.

[B39] Sirotic AC, Coutts AJ (2007). Physiological and performance test correlates of prolonged, high-intensity, intermittent running performance in moderately trained women team sport athletes. Journal of strength and conditioning research / National Strength & Conditioning Association.

[B40] Brenner M, Rankin JW, Sebolt D (2000). The effect of creatine supplementation during resistance training in women. J Strength Cond Res.

[B41] Ledford A, Branch JD (1999). Creatine supplementation does not increase peak power production and work capacity during repetitive Wingate testing in women. J Strength Cond Res.

[B42] Bessman SP, Geiger PJ (1981). Transport of energy in muscle: the phosphorylcreatine shuttle. Science (New York, NY.

[B43] Nelson A, Day R, Glickman-Weiss E, Hegstad M, Sampson B (1997). Creatine supplementation raises anaerobic threshold. FASEB J.

